# Modification of the Interleukin-6 Response to Air Pollution by Interleukin-6 and Fibrinogen Polymorphisms

**DOI:** 10.1289/ehp.0800370

**Published:** 2009-04-06

**Authors:** Petter Ljungman, Tom Bellander, Alexandra Schneider, Susanne Breitner, Francesco Forastiere, Regina Hampel, Thomas Illig, Bénédicte Jacquemin, Klea Katsouyanni, Stephanie von Klot, Wolfgang Koenig, Timo Lanki, Fredrik Nyberg, Juha Pekkanen, Riccardo Pistelli, Christos Pitsavos, Mårten Rosenqvist, Jordi Sunyer, Annette Peters

**Affiliations:** 1 Department of Cardiology, Karolinska Institutet, Stockholm South Hospital, Stockholm, Sweden; 2 Institute of Environmental Medicine, Karolinska Institutet, Stockholm, Sweden; 3 Helmholtz Zentrum München, German Research Center for Environmental Health, Institute of Epidemiology, Munich, Germany; 4 Department of Epidemiology, Local Health Authority, RME, Rome, Italy; 5 Centre for Research in Environmental Epidemiology, Barcelona, Spain; 6 Institut national de la santé et de la recherche médicale U780, Epidemiology and Biostatistics, University Paris-Sud, IFR69, Villejuif, France; 7 Department of Hygiene and Epidemiology, University of Athens Medical School, Athens, Greece; 8 Department of Environmental Health, Harvard School of Public Health, Boston, Massachusetts, USA; 9 Department of Internal Medicine II—Cardiology, University of Ulm Medical Center, Ulm, Germany; 10 Environmental Epidemiology Unit, National Public Health Institute, Kuopio, Finland; 11 AstraZeneca R&D, Mölndal, Sweden; 12 Department of Pneumology, Catholic University, Rome, Italy; 13 First Cardiology Clinic, Ippokration Hospital, University of Athens, Athens, Greece; 14 Municipal Institute of Medical Research, Barcelona, Spain; 15 CIBER in Epidemiology and Public Health, Barcelona, Spain; 16 Department of Health and Experimental Sciences, University Pompeu Fabra, Barcelona, Spain

**Keywords:** air pollution, fibrinogen, gene-environment interaction, interleukin-6, myocardial infarction survivors, polymorphisms

## Abstract

**Background:**

Evidence suggests that cardiovascular effects of air pollution are mediated by inflammation and that air pollution can induce genetic expression of the interleukin-6 gene (*IL6*).

**Objectives:**

We investigated whether *IL6* and fibrinogen gene variants can affect plasma IL-6 responses to air pollution in patients with cardiovascular disease.

**Methods:**

We repeatedly determined plasma IL-6 in 955 myocardial infarction survivors from six European cities (*n* = 5,539). We conducted city-specific analyses using additive mixed models adjusting for patient characteristics, time trend, and weather to assess the impact of air pollutants on plasma IL-6. We pooled city-specific estimates using meta-analysis methodology. We selected three *IL6* single-nucleotide polymorphisms (SNPs) and one SNP each from the fibrinogen α-chain gene (*FGA*) and β-chain gene (*FGB*) for gene–environment analyses.

**Results:**

We found the most consistent modifications for variants in *IL6* rs2069832 and *FBG* rs1800790 after exposure to carbon monoxide (CO; 24-hr average; *p*-values for interaction, 0.034 and 0.019, respectively). Nitrogen dioxide effects were consistently modified, but *p*-values for interaction were larger (0.09 and 0.19, respectively). The strongest effects were seen 6–11 hr after exposure, when, for example, the overall effect of a 2.2% increase in IL-6 per 0.64 mg/m^3^ CO was modified to a 10% (95% confidence interval, 4.6–16%) increase in IL-6 (*p*-value for interaction = 0.002) for minor homozygotes of *FGB* rs1800790.

**Conclusions:**

The effect of gaseous traffic-related air pollution on inflammation may be stronger in genetic subpopulations with ischemic heart disease. This information could offer an opportunity to identify postinfarction patients who would benefit more than others from a cleaner environment and antiinflammatory treatment.

Although air pollution has been recognized as a risk factor for cardiovascular morbidity and mortality (Brook et al. 2004), its mechanism of action remains largely unclear. Inflammation has been proposed as a possible mechanism, and this possibility is supported by experimental models (Donaldson et al. 2003; Niwa et al. 2008; Vogel et al. 2005) and population studies (Rückerl et al. 2006, 2007; Schwartz 2001). A key player in modulating the inflammatory response is interleukin-6 (IL-6), a pleiotropic cytokine shown to be associated with increased risk of cardiovascular morbidity and mortality (Rattazzi et al. 2003).

Levels of IL-6 in plasma may be affected by specific genetic variations of both the gene encoding IL-6 (*IL6*; GenBank accession number M18403) (Bennermo et al. 2004; Burzotta et al. 2001) and genes encoding the fibrinogen protein [α-chain (*FGA*), β-chain (*FGB*), and γ-chain (*FGG*); GenBank accession numbers NM_000508, NM_005141, and NM_021870, respectively] (Mannila et al. 2007; National Center for Biotechnology Information 2009). Because IL-6 principally stimulates fibrinogen production (Dalmon et al. 1993), a possible explanation for increased IL-6 levels in association with certain fibrinogen gene polymorphisms is a positive feedback mechanism where fibrinogen products (fibrin) increase IL-6 production in monocytes and macrophages (Ritchie et al. 1982; Robson et al. 1994; Szaba and Smiley 2002).

Several studies have shown expression of the *IL6* gene to be induced by air pollution (Quay et al. 1998; Vogel et al. 2005; Watterson et al. 2007), but no studies have examined induction of fibrinogen genes or examined variants of the *IL6* gene and how they may possibly lead to differences in individual susceptibility to air pollution. In previous analyses from this study population, genetic polymorphisms of fibrinogen have been associated with increased fibrinogen plasma levels (Jacquemin et al. 2008) and modified the fibrinogen response to ambient particulate matter (PM) (Peters et al. 2009). In light of a positive feedback mechanism of fibrinogen levels on IL-6 levels, polymorphisms of fibrinogen may potentially affect IL-6 levels differently through differences in transcription rate of fibrinogen. Indeed, combinations of polymorphisms in the *IL6* and fibrinogen genes may interact to further increase IL-6 levels.

Myocardial infarction (MI) survivors have a compromised long-term prognosis, making relative risk reduction particularly advantageous. We hypothesized that the moderate effect of air pollution seen on inflammatory markers in previous studies is stronger in MI survivors with specific variants of inflammatory genes such as *IL6* and the fibrinogen genes. We aimed to assess the influence of polymorphisms of the *IL6*, *FGA*, and *FGB* genes on the IL-6 response to air pollution and to explore the influence of gene–gene interactions on this effect.

We present here results of a meta-analysis of six independent studies whose data were collected by a common protocol and that were designed to look at gene–air pollution interactions (Peters et al. 2007)

## Materials and Methods

### Patients

IL-6 concentrations were assessed in the AIRGENE study, a multicenter longitudinal study of MI survivors from six European cities: Athens, Greece; Augsburg, Germany; Barcelona, Spain; Helsinki, Finland; Rome, Italy; Stockholm, Sweden (Peters et al. 2007). Subjects between 35 and 80 years of age who had experienced an MI between 4 months and 6 years before start of the study were recruited through population-based MI registries (KORA MI Registry Augsburg, and registries in Barcelona and Stockholm) or from administrative databases of hospital admissions (Athens, Helsinki, and Rome). Patients with MI or interventional procedures < 3 months before the beginning of the study or with chronic recurring inflammatory diseases such as Crohn’s disease were excluded. MI was defined based on the European Society of Cardiology/American College of Cardiology Committee criteria (Anonymous 2000). Study protocols were approved by local ethics committees, and written consent was obtained from all patients before inclusion in the study.

Patients were invited to participate in six to eight clinical visits between May 2003 and July 2004. The visits were scheduled every 4–6 weeks, on the same weekday and at the same time of the day to minimize the impact of weekly and circadian variation in biological processes and air pollution levels. The average number of visits per patient was 5.8, resulting in 5,813 plasma samples. The subjects recruited were predominantly middle-age men who had survived one MI for > 2 years on average (Table 1).

### Field study

The fieldwork was based on standard operating procedures developed by the AIRGENE study group. At a baseline clinical visit, a single blood sample for DNA analysis was collected and stored at − 80°C until shipment on dry ice for DNA isolation at the laboratory at the Helmholtz Zentrum München—German Research Center for Environmental Health in Neuherberg, Germany. Patients were also characterized at baseline with respect to comorbidities, smoking history, environmental tobacco exposures, socioeconomic status, regular exercise, and alcohol and medication intake. Measurements of blood pressure, body mass index (BMI), total cholesterol, high-density lipoprotein (HDL) cholesterol, glycosylated hemoglobin A1c (HbA1c), and N-terminal proB-type natriuretic peptide (NT-proBNP) were performed.

At each repeated clinical visit, including baseline, a short questionnaire was administered regarding smoking behavior, time of last meal, and a 7-day recall of medication intake. In addition, a blood sample was collected for IL-6 assessment according to previously described procedures (Peters et al. 2007). For quality assurance purposes, 102 randomly selected IL-6 duplicate samples from all centers except Athens were analyzed, showing an average coefficient of variation of 13.8% (from 3.2% in Augsburg to 23.4% in Stockholm).

### Air pollution

Data on hourly means of carbon monoxide (CO), nitrogen dioxide (NO_2_), PM with an aerodynamic diameter ≤ 10 μm (PM_10_) or ≤ 2.5 μm (PM_2.5_), particle number concentration (PNC), air temperature, and relative humidity were collected for each city (Rückerl et al. 2007) and aggregated to 6- and 24-hr running means on the basis of standard procedures (Katsouyanni et al. 1995). Inhalable (PM_10_) and thoracic (PM_2.5_) particles were strongly correlated (overall *r* across cities for consecutive 24-hr means = 0.81), and local combustion-related pollutants were strongly correlated (CO and NO_2_, overall *r* = 0.68; and both also to PNC, overall *r* = 0.67 and 0.74, respectively). We also saw this overall pattern in the single cities, with the exception of Athens and Stockholm, which showed less correlation between CO and PNC.

### Genotyping

We extracted DNA from EDTA (ethylenediaminetetraacetic acid)–anticoagulated blood using a salting out procedure. We selected 10 single-nucleotide polymorphisms (SNPs) for *IL6* and 21 SNPs for *FGA*, *FGB*, and *FGG* and genotyped them as described previously (Peters et al. 2007). Genotyping success rates ranged between 97.9% for *IL6* rs2069832 and 99.8% for *FGB* rs1800790.

### SNP selection

We selected IL6 SNPs that showed an association with IL-6 levels in our sample (*p*-value < 0.05) and were in Hardy-Weinberg equilibrium for gene–environment analyses (Ljungman et al. 2008). Five SNPs met this criterion, but *IL6* rs2069832 and rs1800795 were in very high linkage disequilibrium (LD) (*r*^2^ = 0.99), as were rs1554606 and rs2069845 (*r*^2^ = 0.99). Therefore, one of each pair (rs2069832 and rs1554606) was chosen for further gene–environment interaction analyses. *IL6* rs2069840 was, however, in weak LD (*r*^2^ = 0.31 to 0.35) with the other selected SNPs and might have independent effects. Associations have been seen between IL-6 levels and fibrinogen levels (Mannila et al. 2004), and previous analyses in our study population (Jacquemin et al. 2008) have shown evidence of modification of the effect of fibrinogen polymorphisms on fibrinogen levels by levels of IL-6. Consequently, we selected one SNP each from the genes coding the α-chain (*FGA*, rs2070011) and β-chain (*FGB*, rs1800790) of fibrinogen, which were both associated with fibrinogen levels in the previous study (Jacquemin et al. 2008), based on these results.

Of the 1003 study participants, we restricted all further analyses to 955 (95%) for whom complete information on the five SNPs was available.

### Statistical analysis

We estimated the effect of the SNPs on IL-6 using linear mixed effects models with random subject-specific intercepts, adjusting linearly for the variables age, BMI, pack-years of cigarette smoking, HDL cholesterol, systolic blood pressure, alcohol intake, and logarithm of NT-proBNP and for the categorical variables city, HbA1c, number of earlier MIs, history of heart failure or diabetes, and symptoms of phlegm. SNP genotypes were coded as an ordinal variable by the number of copies of the minor allele (0, 1, 2; denoted 1 1, 1 2, and 2 2 in tables and figures) as described in Ljungman et al. (2008).

For detailed information on model selection procedures, see Rückerl et al. (2007). We estimated the overall effects of air pollutants on IL-6 in city-specific additive mixed models [see Supplemental Material, Table 1 (doi: 10.1289/ehp.0800370.S1 via http://dx.doi.org/)], adjusting for the above-described patient characteristics, time trend, and the weather parameters air temperature and relative humidity to accommodate the different characteristics and meteorologic conditions across Europe, as previously reported (Rückerl et al. 2007). We tested the robustness of the model to exclusion of all time-invariant variables in sensitivity analyses.

Gene–environment interactions were estimated for each SNP by including in these models the main effect of the SNP (additive genetic model), the main effect of the air pollution effect, and the interaction term between air pollution and SNP. We determined the *a priori* exposure window for all gene–environment interaction analyses to be the average exposure during the 24 hr immediately preceding blood withdrawal based on results from previous analyses (Rückerl et al. 2007) and the short half-life of IL-6. Secondary analyses were performed for other time windows of exposure as well as for gene–gene combinations of polymorphisms showing effect modification. We explored consistency of patterns of association in city-specific analyses. In assessing the contributions of the different exposure windows to the interaction effect, we also performed analyses for the subjects carrying at least one risk allele using distributed lags, thus allowing for independence of effects for each time window of exposure in a common model.

Effect estimates by genotypes are presented together with 95% confidence intervals (CIs). Because the study of gene–environment interactions was of an exploratory nature, we did not attempt to correct for multiple comparisons for these analyses. Estimates are presented for an increase of the pollutants by a one interquartile increase in pollutant exposure range and expressed as a relative change of the overall geometric mean in IL-6. We tested the assumption of an additive genetic effect by using an indicator variable for the heterozygote genotype in the model. For overall results, we pooled city-specific estimates using meta-analysis methodology (van Houwelingen et al. 2002). Heterogeneity of effect estimates was assessed with a chi-square test with 5 degrees of freedom. If the *p*-value was < 0.1, random effect models were applied for combining effect estimates.

## Results

Mean IL-6 ranged from 2.5 pg/mL in Augsburg to 3.5 pg/mL in Barcelona (Table 1). The consecutive 24-hr mean levels of CO ranged from 0.29 mg/m^3^ in Stockholm to 1.48 mg/m^3^ in Athens. The within-interquartile difference ranged from 0.08 in Helsinki to 0.73 in Athens.

Less than 5% of the subjects were homozygotes for the minor allele of *FGB* rs1800790, whereas 9–19% of subjects were homozygotes for minor allele of the *IL6* and *FGA* polymorphisms (Table 2). Genotype frequencies differed among the cities for all SNPs (chi-square test, *p* < 0.03), displaying a north–south gradient, with the minor alleles of *IL6* rs2069832 and *IL6* rs2069845 having higher frequencies in the northern cities and the minor alleles of *IL6* rs2069840 having higher frequencies in the southern cities. The fibrinogen SNPs did not show such a pattern (data not shown).

### Overall effect of genotype on IL-6 levels

Table 2 shows the associations of SNPs with an increased plasma level of IL-6 in this data set. The minor alleles of *IL6* rs2069832 and *IL6* rs2069845 and the major allele of *IL6* rs2069840 were significantly associated with an increased plasma level (at *p* < 0.05) (Ljungman et al. 2008). The minor allele of *FGA* rs2070011 showed nonsignificant association to increased plasma levels of IL-6, whereas *FGB* rs1800790 polymorphism showed no clear association to plasma IL-6. We found no evidence of deviation from the additive model for these SNPs (*p*-value for heterozygote indicator variable > 0.05).

### Overall effects of air pollution on IL-6 levels

Elevated concentrations of CO averaged during the 0–24 hr preceding blood sampling were not significantly associated with increased plasma IL-6 levels (Table 3), but elevated concentrations during 6–11 hr intervals preceding sampling were associated (2.2% change of overall mean IL-6; 95% CI, 0.3–4.2%). For other pollutants, NO_2_ levels averaged over the 0–24 hr preceding blood sampling showed associations with an increase in plasma IL-6 (Table 3), and PNC levels showed significant associations for both 6–11 hr and 12–17 hr exposure windows preceding sampling [1.8% change (95% CI, 0.2–3.5%) and 2.6% change (95% CI, 0.8–4.5%), respectively].

### Genotype modification of IL-6 response (24-hr exposure windows)

Subjects with the homozygous major allele genotypes for all three *IL6* polymorphisms showed larger IL-6 responses to increased CO, but only for for *IL6* rs2069832 was a similar genetic inter-action seen with increased NO_2_ (Table 4).

Subjects with the homozygote minor allele genotype of *FGB* rs1800790 showed both a larger and clearer effect modification for the IL-6 response to increased CO compared with the *IL6* SNPs. The overall effect of a 0.8% increase in IL-6 per 0.34 mg/m^3^ increase of CO in the preceding 0–24 hr seemed to be confined to individuals carrying the minor allele of *FGB* rs1800790. In the 4% of the study sample that were homozygous with this allele, the corresponding CO effect on IL-6 was a 4.5% increase. We saw similar magnitudes of effect modification for NO_2_, but the effect modification pattern was not statistically significant. The *FGA* SNP did not modify the response to air pollution. These results were robust to sensitivity analysis excluding all time-invariant variables from the final model and only maintaining time trend and meteorology (data not shown).

**Figure 1:**
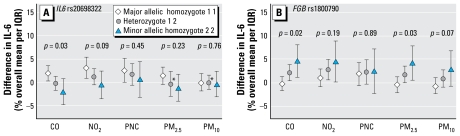
Modification by *IL6* rs2069832 (*A*) and *FGB* rs1800790 (*B*) genotypes of IL-6 response to increased 24-hr average ambient CO, NO_2_, ultrafine PM (PNC), and PM_2.5_ immediately preceding blood withdrawal (1, major allele; 2, minor allele). Error bars indicate 95% CIs; *p*-values are for significance of the inter action term. Interquartile ranges: CO, 34.0 mg/m^3^; NO_2_, 15.9 μg/m^3^; PNC, 11,852/cm^3^; PM_2.5_, 11.0 μg/m^3^; PM_10_, 17.4 μg/m^3^. *Heterogeneity of the city-specific effect estimates with a *p*-value < 0.1.

For PM pollutants, we found a similar pattern of effect modification of *FGB* rs1800790 for increased levels of PM_2.5_ and PM_10_ during the preceding 0–24 hr, but with less precision of effect for PM_10_ (Figure 1). For ultrafine particles measured by PNC and for *IL6* rs2069832, the pattern was less clear.

**Figure 2:**
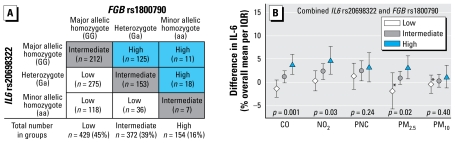
(*A*) Construction of gene–gene interaction variable based on relative change in plasma IL-6 in univariate gene–environment interaction analysis of polymorphisms *IL6* rs2069832 and *FGB* rs1800790. (*B* ) Modification by gene–gene interaction of *IL6* rs2069832 and *FGB* rs1800790 of IL-6 response to increased preceding ambient 24-hr air pollutant averages. Error bars indicate 95% CIs, and *p*-values are for significance of the interaction term. Interquartile ranges: CO, 0.34 mg/m^3^; NO_2_, 15.9 μg/m^3^; PNC, 11,852/cm^3^; PM_2.5_, 11.0 μg/m^3^; PM_10_, 17.4 μg/m^3^. *Heterogeneity of the city-specific effect estimates with a *p*-value < 0.1.

When we classified different combinations of the *IL6* rs2069832 and *FGB* rs1800790 genotypes according to number of risk alleles into low, intermediate, and high risk (Figure 2A), we found significant modification of the IL-6 response to an increase in the preceding 0–24 hr average of CO as well as both NO_2_ and PM_2.5_ (Figure 2B). Subjects with ≥ three high-risk alleles from both SNPs (154 subjects) had the largest IL-6 response.

### Genotype modification of IL-6 response (6-hr exposure windows)

**Figure 3:**
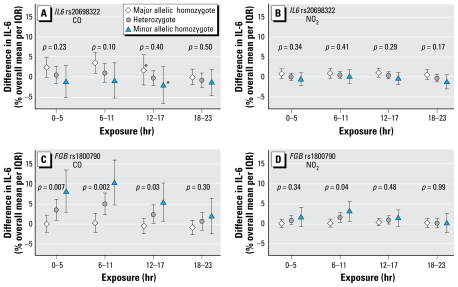
Modification by *IL6* rs2069832 and *FGB* rs1800790 genotypes of IL-6 response to increased ambient CO and NO_2_ in association with 6-hr exposure windows preceding blood sampling. Error bars indicate 95% CIs, and *p*-values are for significance of the interaction term. Interquartile ranges for 6-hr means: CO, 0.64 mg/m^3^; NO_2_, 11.5 μg/m^3^. *Heterogeneity of the city-specific effect estimates with a *p*-value < 0.1.

Analyses of 6-hr exposure windows during the 24 hr immediately preceding blood sampling showed the clearest effect modification of the IL-6 response to CO within the three preceding 6-hr windows (Figure 3). We found greatest effect modification for *FGB* rs1800790 for increased CO during the 6–11 hr preceding sampling. The overall effect of a 2.2% increase in IL-6 per 0.64 mg/m^3^ increase of CO in this time window seemed again to be confined to individuals carrying the minor allele of *FGB* rs1800790, with a 10% (95% CI, 4.6–16%) increase in homozygotes (4% of study sample). In homozygotes for the major allele of IL6 rs2069832 (36% of the study sample), the corresponding increase was 3.6% (95% CI, 1.0–6.2%). Differences in the NO_2_ (Figure 3) and PM_2.5_ (data not shown) effects across genetic subgroups for different 6-hr exposure windows were similar but weaker. The distributed lag analyses restricted to subjects with at least one minor allele of *FGB* rs1800790 (GA or AA, 37% of study sample) or one major allele of *IL6* 2069832 (GA or AA, 83% of study sample) for 6-hr and 24-hr time windows showed associations with increased IL-6 for increases in CO for the 0–5 hr and 6–11 hr preceding sampling [see Supplemental Material, Figure 1 (doi: 10.1289/ehp.0800370.S1)]. The IL-6 increase seen in minor allele holders of *FGB* rs1800790 for 0–23 hr exposure of CO seemed to be mainly driven by exposure in the 6–11 hr time window. We found a similar but weaker pattern for 0–23 hr exposure in major allele holders of *IL6* rs2069832.

### City-specific modification of IL-6 response to CO and NO_2_

In a comparison of city-specific analyses for *IL6* rs2069832 [see Supplemental Material, Figure 2 (doi: 10.1289/ehp.0800370. S1)], modification of the IL-6 response to preceding 0–24 hr average of increased CO and NO_2_ showed consistency between the pollutants, with the clearest effect modification seen in Helsinki, Barcelona, and Athens. For *FGB* rs1800790 [see Supplemental Material, Figure 3 (doi: 10.1289/ehp.0800370.S1)], we found the strongest effect modification in Athens and Augsburg, and the effects were consistent for both pollutants except in Stockholm. Although we found variation across cities because of reduced power in the city-specific analyses, we found no significant heterogeneity of city- specific effects (*p*-value > 0.1).

## Discussion

We found evidence of gene–environment interaction where the SNPs *IL6* rs2069832 and *FGB* rs1800790 modified the effects of air pollution on IL-6 levels in MI survivors. All pollutants showed similar patterns of effect, but results were most apparent for CO. The influence of CO on IL-6 levels was strongest in subjects with the major allele of *IL6* rs2069832 and the minor allele of *FGB* rs1800790, and the combination of these two genetic variants, present in 16% of subjects, resulted in a significant gene–gene–environment interaction, potentially indicating a higher risk for health effects from air pollution in these patients with ischemic heart disease. We found similar magnitudes of effect modification with respect to air pollution for *IL6* rs2069840 and *IL6* rs2069845, but we found no effect modification for *FGA* rs2070011.

We found small effects of air pollution on IL-6 levels in the overall population. The groups defined by the minor allele of *FGB* rs1800790, and to a lesser extent by the major allele of *IL6* rs2069832, seem to be the driving force between the associations seen, suggesting a restriction of the effect of air pollution to groups of MI survivors of specific genotypes.

NO_2_ was the pollutant with the most convincing effects on IL-6 levels with respect to the a priori specified time window of exposure (0–24 hr) when considering the overall population, regardless of genotype. Modification of air pollution effects by SNPs was, however, clearest for CO, although the other pollutants in general followed a similar pattern. CO has been proposed as a probable culprit to cardiovascular effects of air pollution (Dales 2004; McGrath 2000) in addition to PM. In this study we did not have sufficient power to more specifically identify the most important air pollution component or source, and we acknowledge that we studied a mixture of correlated pollutants. Ambient CO levels measured using fixed monitors have been shown to correlate better to personal exposure to PM rather than to personal exposure to CO (Sarnat et al. 2005; Schwartz et al. 2007), so although sensitivity analyses adjusting for the level of PM_2.5_ in the models showed support for an independent effect of CO (data not shown), we can not be sure of a specific CO effect. Secondary analyses showed that the interaction effect was clearest and strongest during the first 24 hr and particularly 6–11 hr preceding blood withdrawal, consistent with the short half-life of IL-6. The early effect was further supported by the distributed lag analyses of the subjects carrying the risk allele for *IL6* rs2069832 and *FGB* rs1800790. Given the strong correlation of 6-hr exposure windows, it is probable that effects seen for exposure window 12–17 hr and possibly also 0–5 hr reflect this correlation rather than independent effects of these exposure windows.

Although *FGB* rs1800790 displayed an interaction effect with CO on IL-6 levels, we found no effect of the polymorphism on IL-6 in these patients when not taking air pollution into account. A previous study of the same population (Jacquemin et al. 2008) investigating the effect of fibrinogen polymorphisms on fibrinogen plasma levels found no interaction effect with high levels of IL-6 on fibrinogen levels for this polymorphism, in contrast to *FGA* rs2070011. However, a follow-up study (Peters et al. 2009) showed that *FGB* rs1800790 modified the effect of a 5-day average exposure to ambient PM on circulating fibrinogen levels. The production of fibrinogen is stimulated by IL-6, but there is also evidence both *in vivo* (Szaba and Smiley 2002) and *in vitro* (Robson et al. 1994) suggesting a positive feedback mechanism whereby fibrinogen products in turn stimulate IL-6 production and release from monocytes. Although this might suggest that the increased IL-6 levels seen in MI survivors with a minor allelic genotype of *FGB* rs1800790 are mediated via increased fibrinogen levels, the effects on IL-6 levels occurred within 12 hr rather than after 5 days.

In this study, subjects with major alleles of *IL6* rs2069832 and rs2069845 had lower IL-6 concentrations than did those with minor alleles, but they seemed to react more strongly to CO exposure than did minor allele holders. The reason for this pattern is unclear. Our previous study (Ljungman et al. 2008) revealed a tendency of a greater within-individual variability for major allele holders compared with minor allele holders. Taken together, this may indicate that major allele holders are more likely to respond with inflammation to stimuli such as air pollution but from a lower basal level of IL-6.

Air pollution has been associated in epidemiologic studies with the onset of MIs in both previously healthy subjects and cardiac patients (Peters et al. 2001; Zanobetti and Schwartz 2005). In addition, human experimental (Mills et al. 2007) and epidemiologic (Chuang et al. 2008) studies have demonstrated an effect of air pollution on ST-segment changes in electrocardiograms indicating the triggering of acute ischemia. The gene–environment interaction observed for these two SNPs in our study emphasizes that certain genetic subgroups of cardiac patients may react more strongly toward air pollution than do others. Similar genetic interaction with air pollutants has been seen in human and animal models for lung disease (Kleeberger 2003; London 2007) and oxidative stress (Romieu et al. 2006; Schwartz et al. 2005). Although both the *IL6* and *FGB* genes are probable candidates affecting the levels of the known cardiovascular biomarker IL-6, the actual polymorphisms responsible for the observed effect modification may be other strongly correlated SNPs or combinations of SNPs. Indeed, in our population, *IL6* rs2069832 has very high LD with *IL6* rs1800795, a functional SNP located in the promoter region. The reason for focusing on *IL6* rs2069832 instead of *IL6* rs1800795 was that the overall pooled effects of *IL6* rs1800795 on IL-6 levels showed slightly more heterogeneity across cities (Ljungman et al. 2008). To confirm the effects of the studied polymorphisms, functional analyses are required, and as our analyses suggest, the nature of possible gene–gene interaction effects in combinations of SNPs possibly in different genes are of interest. In order to verify whether these data have any prognostic significance, follow-up of clinical ischemic events has to be performed.

### Strengths and limitations

We conducted this study with data from six European cities, thereby providing six independent samples for testing gene–environment interactions. We focus here on genes that, applying a meta-analytical approach across all cities, were associated with gene–environment interactions.

The different cities, however, reflect not only a large variation of air pollution exposure, which was desirable, but also other differences possibly confounding our results. To control for this variation, we applied a two-step approach, starting with city-specific models and thereafter pooling the results and checking for evidence of heterogeneity. This test, however, suffers from low power, and the interaction effects showed some disparity across cities. We found clearer effects in Athens and Barcelona than in Helsinki or Stockholm. The southern European cities showed the highest mean levels and the greatest variability in CO levels, whereas Stockholm and Helsinki showed very little variation and considerably lower mean levels. A sensitivity analysis excluding Athens did reduce the effect estimates for CO and increased the *p*-values of interaction, whereas results for NO_2_ and PM_2.5_ were essentially unchanged (data not shown). However, we present here a pooled analysis of six independently conducted studies based on a common protocol. Therefore, we regard the *a priori* planned analyses as valid, which include all cities, and especially those with the large exposure contrasts and therefore more power. By and large, the effects for CO were corroborated by those for NO_2_. The remaining differences might be attributed to possible differences in measurement error for the pollutants, as well as possible differences in how representative the fixed monitors are of urban background levels of the various pollutants. The low-risk allele showed point estimates below zero for the *IL6* SNP and the combined SNPs in relation to increased CO, for which we have no clear explanation, but because we found no such tendency for the same genotypes in relation to NO_2_, this may be a chance finding. The temporal relationship between exposure and effect within 12 hr generally fits the expected rise and fall of IL-6 response in the bloodstream. A fair amount of measurement error was seen for IL-6, but we expect that this would not be associated with air pollution levels and therefore would lead to nondifferential misclassification, potentially attenuating our results.

We had expected a stronger main effect of CO and NO_2_ on IL-6 levels, but because air pollution is strongly linked to cardiovascular mortality, survivors of MI—although at high absolute risk for future cardiovascular events—may be less susceptible to the effects of air pollution in a relative sense than the general population. Nevertheless, our results indicate that groups of these individuals identified by specific genotypes are at increased risk of a systemic inflammatory response in association with exposure to continuous ambient air pollution. Should this increase in IL-6 be confirmed to have prognostic implications, these individuals could be identified and protected with counseling such as avoidance of traffic or going outdoors at periods of high risk, for example, within the first month after an MI or during highly polluted days. Furthermore, they might be helped by more rigorous medication.

## Conclusion

The results support our hypothesis that the effect of air pollution on inflammation may be stronger in certain genetic subpopulations of cardiac patients. Polymorphisms in the *IL6* genes modified the IL-6 response when exposed to increased air pollution in the 6–11 hr period preceding sampling, consistent with biological expectation. The role of *FGB* rs1800790 also seemed important, although the rapid effect was unexpected. Combining these polymorphisms also showed a gene–gene interaction, possibly implicating that one-sixth of the population of MI survivors is at particular risk for effects of air pollution. Whether these differences in air-pollution–mediated inflammation have any prognostic importance remains to be clarified, as well as the possible role of these polymorphisms in air pollution effects on earlier stages of cardiovascular morbidity.

## Figures and Tables

**Table 1:** Data on patient characteristics, IL-6 measurements, and air pollution concentrations in the AIRGENE study.

Variable	Helsinki	Stockholm	Augsburg	Rome	Barcelona	Athens	Total
No.	182	193	187	132	160	101	955
Sex (% male)	69.8	71.0	81.8	87.1	84.4	86.1	79.0
Age (years)^a^	64.4 (45–78)	64.0 (38–76)	61.9 (39–76)	62.5 (39–79)	62.1 (37–81)	54.5 (38–75)	62.2 (37–81)
BMI (kg/m^2^)^a^	28.8 (19.1–48.9)	27.6 (17.5–43.3)	28.9 (19.1–48.4)	27.7 (19.0–39.4)	28.8 (19.4–43.5)	28.8 (20.9–46.4)	28.4 (17.5–48.9)
HDL/total cholesterol^a^	0.3 (0.1–0.6)	0.3 (0.1–0.6)	0.3 (0.1–0.5)	0.2 (0.1–0.5)	0.3 (0.1–0.5)	0.2 (0.1–0.6)	0.3 (0.1–0.6)
HbA1c (%)^a^	5.9 (4.7–9.2)	5.1 (3.8–9.9)	5.6 (4.7–9.8)	5.4 (2.8–8.7)	5.1 (3.8–9.8)	5.9 (3.7–10.5)	5.5 (2.8–10.5)
NT-proBNP (pg/mL)^a^	392.9 (10.8–3,799)	330.4 (14.0–3,348)	482.5 (12.5–9,308)	405.0 (19.1–6,174)	380.6 (11.3–7,057)	314.9 (11.0–4,855)	389.1 (10.8–9,308)
Current smokers (%)	1.7	0.5	0	9.1	13.8	38.6	8.1
Pack-years (cigarettes only)^a^	9.2 (0–65.0)	12.2 (0–73.8)	15.2 (0–205.2)	22.0 (0–171.8)	28.4 (0–193.2)	35.6 (0–174.0)	18.8 (0–205.2)
Time between last MI and study start (years)^a^	2.7 (0.6–5.8)	2.3 (0.6–3.9)	2.1 (0.5–3.4)	2.7 (0.4–6.0)	2.2 (0.4–5.9)	2.4 (0.5–5.0)	2.4 (0.4–6.0)
First MI (%)	81.9	86.5	88.2	87.1	85.6	81.2	85.3
Self-reported history^b^							
Arrhythmia (%)	1.7	1.8	1.8	1.8	1.9	1.8	1.8
Chronic bronchitis (%)	1.8	1.8	1.9	1.9	2.0	2.0	1.9
Hypertension (%)	1.5	1.5	1.5	1.4	1.5	1.4	1.5
Blood samples (*n*)	1,081	1,146	1,067	728	1,059	458	5,539
IL-6 (pg/mL)^c^	3.1 (0.9–19.7)	2.6 (0.5–24.4)	2.5 (0.6–11.8)	3.0 (1.0–61.4)	3.5 (0.8–28.5)	3.0 (0.8–22.4)	3.0 (0.5–61.4)
Air pollution measurement (study period)^d^	5 Sep 2003 to 2 Jun 2004	30 Aug 2003 to 24 June 2004	14 May 2003 to 24 Feb 2004	20 Sep 2003 to 15 Jul 2004	30 Aug 2003 to 16 Jun 2004	8 Sep 2003 to 30 Jul 2004	
CO (mg/m^3^)	0.31 (0.26–0.34)	0.29 (0.25–0.34)	0.58 (0.43–0.66)	1.40 (1.02–1.66)	0.59 (0.45–0.70)	1.48 (0.95–1.68)	0.78 (0.56–0.90)
NO_2_ (μg/m^3^)	28.6 (20.5–34.6)	18.4 (12.7–22.9)	40.0 (32.7–46.5)	67.0 (56.7–76.3)	50.5 (39.3–60.4)	50.2 (42.0–59.0)	42.4 (34.0–49.9)
PM_2.5_ (μg/m^3^)	8.2 (4.7–10.4)	8.8 (6.0–10.3)	17.4 (12.2–21.2)	24.6 (14.0–30.7)	24.2 (13.5–29.7)	23.0 (14.9–29.1)	17.7 (10.9–21.9)
PM_10_ (μg/m^3^)	17.1 (10.5–20.7)	17.8 (11.0–21.7)	33.1 (22.0–42.7)	42.1 (30.6–49.9)	40.7 (25.1–49.2)	38.5 (27.1–46.4)	31.6 (21.1–38.4)
PNC (per cm^3^)	8,534 (5,834–10,519)	9,748 (7,247–11,625)	11,876 (7,085–14,440)	35,450 (21,094–46,963)	18,133 (10,492–24,278)	20,590 (11,872–26,913)	17,388 (10,604–22,456)
Pearson correlation							
CO							
NO_2_	0.76	0.56	0.53	0.75	0.70	0.55	0.69
PM_2.5_	0.38	0.53	0.61	0.70	0.26	0.45	0.55
PM_10_	0.25	0.30	0.60	0.71	0.30	0.17	0.47
PNC	0.60	0.23	0.77	0.87	0.64	0.25	0.67

a Mean (range).

b Ever diagnosed by a physician.

c Mean (range) of the subject-specific means based on three to eight repeated measurements.

d Mean (25th–75th percentile) for consecutive 24-hr means across study period.

**Table 2:** SNPs and SNP–IL-6 association: characteristics of the analyzed SNPs of *IL6*, *FGB*, and *FGA* and their association with plasma IL-6 concentrations.

SNP	Functional region	Alleles		Genotype frequency [% (*n*)]			Difference in IL-6^a^ level ll [% overall mean: effect of two alleles (2 2 vs. 1 1)]
		Major (1)/Minor (2)	Minor allele frequency [% (*n*)]	Homozygote of major allele (1 1)	Heterozygote (1 2)	Homozygote of minor allele (2 2)	
*IL6*							
rs2069832	Intron	G/A	40 (768)	36 (696)	47 (892)	17 (322)	13.4 (3.7 to 24.0)
rs2069840	Intron	C/G	31 (602)	46 (884)	45 (850)	9 (176)	− 9.6 (− 17.2 to − 1.3)
rs2069845	Intron	A/G	43 (820)	33 (632)	48 (918)	19 (360)	14.6 (4.7 to 25.4)

*FGA*							
rs2070011	Promoter	C/T	39 (744)	39 (738)	45 (858)	16 (314)	8.1 (− 0.5 to 17.5)

*FGB*							
rs1800790	Promoter	G/A	20 (386)	63 (1,210)	33 (628)	4 (72)	− 5.6 (− 14.7 to 4.4)

a Effect estimates and 95% CIs from linear mixed model analysis with log IL-6 as outcome, with a random effect for subject to account for repeated measurements, using an additive genetic model for the genotypes, and adjusted for age, city, glycosylated hemoglobin, number of earlier MIs, earlier diagnosed heart failure or diabetes, symptoms of phlegm, BMI, pack-years of cigarette smoking, HDL cholesterol, systolic blood pressure, alcohol intake, and log NT-proBNP.

**Table 3:** Effect of 24-hr interquartile range increase in air pollution on IL-6 plasma levels.

Air pollutant response without genetic covariate	Interquartile range increase	Change of IL-6^a^ (% of overall mean per interquartile range increase)	*p*-Value
CO	0.34 mg/m^3^	0.8 (− 0.5 to 2.1)	0.22
NO_2_	15.9 μg/m^3^	1.7 (0.2 to 3.4)	0.03
PNC	11,852/cm^3^	1.9 (− 0.2 to 4.0)	0.07
PM_2.5_	11.0 μg/m^3^	0.6 (− 0.8 to 2.0)	0.40
PM_10_	17.4 μg/m^3^	0.0 (− 1.3 to 1.3)	1.0

a Inverse-weighted sum of city-specific estimates and 95% CIs from linear regression using log IL-6 concentrations as outcome, with a random effect for subject, using the 24-hr average of air pollutants fitted to the time of IL-6 measurement and adjusting for age, city, BMI, log NT-proBNP, HDL cholesterol, systolic blood pressure, number of MIs, glycosylated hemoglobin, pack-years of cigarette smoking, alcohol intake, history of arrhythmias, heart failure, bronchitis, season, apparent temperature, relative humidity, and hour of visit in city-specific models.

**Table 4:** SNP–environment interactions: association between 24-hr average of CO or NO_2_ and plasma IL-6 levels by genotype for the analyzed polymorphisms in the genes *IL6*, *FGA*, and *FGB*.

			Change of IL-6^a^ (% of overall mean per interquartile range increase of pollutant)	
SNP	Genotype^b^		CO	NO_2_
*IL6* rs2069832	1 1		2.0 (0.3 to 3.6)	3.1 (0.9 to 5.4)
	1 2		− 0.2 (− 1.7 to 1.3)	1.2 (− 0.5 to 3.0)
	2 2		− 2.0 (− 4.7 to 0.8)	− 0.5 (− 3.5 to 2.5)
		*p*-Value	0.03	0.09

*IL6* rs2069840	1 1		2.0 (0.3, 3.8)	1.8 (− 0. 4 to 4.0)
	1 2		0.4 (− 0.9, 1.7)	1.7 (0.0 to 3.4)
	2 2		− 1.2 (− 3.4, 1.1)	1.7 (− 1.5 to 4.9)
		*p*-Value	0.04	0.93

*IL6* rs2069845	1 1		1.9 (0.2 to 3.5)	2.8 (0.5 to 5.1)
	1 2		− 0.1 (− 1.5 to 1.4)	1.3 (− 0.4 to3.1)
	2 2		− 1.6 (− 4.3 to 1.2)	0.2 (− 2.6 to 3.2)
		*p*-Value	0.31	0.26

*FGA* rs2070011	1 1		1.0 (− 0.7 to 2.7)	2.1 (− 0.1 to 4.3)
	1 2		0.7 (0.6 to 2.0)	1.6 (− 0.1 to 3.3)
	2 2		0.4 (− 1.9 to 2.7)	1.1 (− 1.8 to 4.1)
		*p*-Value	0.64	0.64

*FGB* rs1800790	1 1		− 0.2 (− 1.8 to 1.3)	1.0 (− 0.9 to 2.9)
	1 2		2.1 (0.4 to 3.8)	2.8 (0.6 to 5.0)
	2 2		4.5 (1.1 to 8.0)	4.4 (0.1 to 8.9)
		*p*-Value	0.02	0.19

a Inverse-variance weighted sum of city-specific estimates and 95% CIs from linear regression using IL-6 concentrations as outcome and an additive genetic model for the genotype, with a random effect by subject, using the 24-hr average of CO or NO_2_ fitting to the time of IL-6 measurement, including a genotype–CO interaction adjusted for age, city, BMI, log NT-proBNP, HDL cholesterol, systolic blood pressure, number of MIs, glycosylated hemoglobin, pack-years of cigarette smoking, alcohol intake, history of arrhythmias, heart failure, bronchitis, season, apparent temperature, relative humidity, and hour of visit in city-specific models. Interquartile range increase: CO, 0.34 mg/m^3^; NO_2_, 15.9 μg/m^3^.

b 1, major allele; 2, minor allele.
